# Refeeding Syndrome: A Literature Review

**DOI:** 10.1155/2011/410971

**Published:** 2010-08-25

**Authors:** L. U. R. Khan, J. Ahmed, S. Khan, J. MacFie

**Affiliations:** The Combined Gastroenterology Research Unit, Scarborough Hospital, Woodland Drive, Scarborough YO12 6QL, UK

## Abstract

Refeeding syndrome (RFS) describes the biochemical changes, clinical manifestations, and complications that can occur as a consequence of feeding a malnourished catabolic individual. RFS has been recognised in the literature for over fifty years and can result in serious harm and death. Crude estimates of incidence, morbidity, and mortality are available for specific populations. RFS can occur in any individual but more commonly occurs in at-risk populations. Increased awareness amongst healthcare professionals is likely to reduce morbidity and mortality. This review examines the physiology of RFS and describes the clinical manifestations. A management strategy is described. The importance of a multidisciplinary approach is emphasized.

## 1. Introduction

RFS is well recognised. It occurs after the reintroduction of feeding after a period of starvation or fasting [1]. RFS describes a series of metabolic and biochemical changes that occur as a consequence of reintroduction of feeding after a period of starvation or fasting. This unfavorable metabolic response causes nonimmune-mediated harm to the body and can be mild, moderate, or severe. Although, the physiology and pathophysiology are well known, the circumstances under which RFS occurs, the clinical manifestations, and the management of these patients are less clear [2, 3].

### 1.1. Methods

A PubMed search for the terms “refeeding syndrome” AND “hypophosphataemia” generated two hundred and seven separate articles. There were no randomized controlled trials identified.

### 1.2. Physiology

With food in abundance, carbohydrates provide for most of our energy requirements. Glucose, the principal product of carbohydrate digestion, is actively cotransported along with sodium at the intestinal brush border against a concentration gradient. Glucose enters the portal circulation by facilitated diffusion and blood sugar levels rise. This stimulates the release of the peptide hormone insulin from pancreatic islet cells. Insulin secretion has several effects. It promotes glucose uptake and storage (glycogenesis), inhibits the breakdown of fats (lipolysis), and increases cellular uptake of potassium. When glycogen storage capacity is exceeded, lipogenesis occurs with nonoxidised glucose being converted to fat and stored as triglycerides in adipose tissue. Together, the consequence is for blood glucose levels to fall with a concomitant reduction in insulin secretion [4].

 With starvation, levels of glucose begin to fall within 24 to 72 hours. This results in the release of the peptide hormone glucagon and a reduction in insulin secretion [10]. Glucose levels are maintained by glycogenolysis but glycogen stores rarely last more than 72 hours [11]. Glucose homeostasis is essential because certain tissues, such as brain, erythrocytes, and cells of the renal medulla are obligate glucose users [12]. These demands for glucose are met by the process of gluconeogenesis by which noncarbohydrate sources are metabolized to glucose. The most important of these is the muscle protein alanine. In addition, fatty acid oxidation in liver hepatocytes generates ketone bodies. These are converted to acetyl-coenzyme-A generating energy via the Kreb's cycle. Further energy production from lactate and pyruvate (the products of glycolysis) and amino acids occurs via the Cori cycle [4]. In summary, metabolic adaptation occurs to ensure survival on fat fuel economy [13]. There is a resultant loss of body fat and protein and an accompanying depletion of potassium, phosphate, and magnesium [14, 15]. Homeostatic mechanisms maintain serum concentrations of these ions at the expense of intracellular stores. Serum levels may remain normal despite a marked reduction in total body levels. 

The reintroduction of nutrition to a starved or fasted individual results in a rapid decline in both gluconeogeneis and anaerobic metabolisms [13]. This is mediated by the rapid increase in serum insulin that occurs on refeeding [10]. Insulin stimulates the movement of extracellular potassium, phosphate, and magnesium to the intracellular compartment. Depleted intracellular stores and a large concentration gradient ensure a rapid fall in the extracellular concentration of these ions [16, 17]. Osmotic neutrality must be maintained resulting in the retention of sodium and water [18]. Reactivation of carbohydrate-dependent metabolic pathways increases demand for thiamine, a cofactor required for cellular enzymatic reactions [19, 20]. The deficiencies of phosphate, magnesium, potassium, and thiamine occur to varying degrees and have different effects in different patients [5]. Some, such as chronic alcohol abusers or those with long-term starvation, are more vulnerable to the metabolic consequences of mineral or elemental deficiencies [21–28]. This explains why RFS is not defined by a clear set of signs and symptoms but is considered an arbitrary term referring to a wide spectrum of biochemical abnormalities and clinical consequences [5] (Table 2).

### 1.3. What Is Refeeding Syndrome?

First reports of the syndrome appeared in the 1950s after observations of malnourished prisoners of war who developed cardiac and neurological symptoms soon after the recommencement of feeding [31, 32]. There is no internationally agreed definition of RFS [6]. In 2001 Crook et al. referred to a syndrome of severe electrolyte and fluid shifts associated with metabolic abnormalities in malnourished patients undergoing refeeding, whether orally, enterally, or parenterally [1].

As there is no strict definition, it is not surprising that the incidence of RFS is unclear. Robust epidemiological studies are lacking in part due to the absence of accepted diagnostic criteria or internationally agreed guidelines for detecting RFS [5]. Most published data from prospective and retrospective case series do not reflect overall incidence.

Hypophosphataemia is the adopted surrogate marker for diagnosing RFS though low serum phosphate is not pathognomonic [33]. Estimates of hypophosphataemia in those at risk of RFS are high [2, 7, 34]. In a prospective study of sixty-two patients in the intensive care unit refed after being starved for 48 hours, twenty-one patients (34%) experienced refeeding hypophosphataemia. There was an association with low prealbumin concentration [7]. In a separate study of one hundred and six patients with histologically confirmed cancer, the incidence was 25% [34]. Hypophosphataemia is uncommon in the hospitalized patient population, occurring in 2% of all requests received for serum phosphate determination in one institution over an eighteen-month period [33]. There are limitations to relying on low serum phosphate and levels may be normal in patients with multiorgan failure or in the presence of impaired renal function.

An alternative to the lack of reliable RFS incidence data is to examine the prevalence of those at risk of RFS. A consensus exists in the literature that prevention is preferable to treating established RFS. Therefore, estimating the prevalence of those at risk might assist in understanding the potential scale of RFS. The predominant risk factor for RFS is malnutrition. The report of the British Association of Parenteral and Enteral Nutrition (BAPEN, 2008) estimated in the UK that there were more than three million people who are either malnourished or at risk of malnutrition whilst the British Dietetic Association estimated a 20% to 60% risk of malnutrition in patients being admitted to hospital. In 2005 Hise et al. estimated that 30% to 50% of hospitalized patients are malnourished [35]. Morley in 2002 estimated the prevalence of malnutrition is 1% to 15% in patients attending outpatient, 25% to 60% in the institutionalized patients, and it is 35% to 65% in hospitalized patients [36]. A hospital-based study screened 32,837 patients finding nearly one fifth were severely undernourished or at risk for undernutrition and that the risk was directly related to age [37]. Malnutrition is a problem in many different disease groups, including cancer (5–80%), neurology (4–66%), elderly (0–85%), surgical/critical illness (0–100%), respiratory disease (5–60%), gastrointestinal and liver disease (3–100%), HIV/AIDS (8–98%), and renal disease (10–72%) [30] (Table 2). These data underscore the significant possibility for RFS and highlight the link between comorbidity, nutritional status, and RFS.

## 2. Clinical Manifestations

Symptoms of RFS are variable, unpredictable, may occur without warning, and may occur late. Symptoms occur because changes in serum electrolytes affect the cell membrane potential impairing function in nerve, cardiac, and skeletal muscle cells. The variable clinical picture in RFS reflects the type and severity of biochemical abnormality present. With mild derangements in these electrolytes, there may be no symptoms. More often, the spectrum of presentation ranges from simple nausea, vomiting, and lethargy to respiratory insufficiency, cardiac failure, hypotension, arrhythmias, delirium, coma, and death. Clinical deterioration may occur rapidly if the cause is not established and appropriate measures not instituted. Low serum albumin concentration may be an important predictor for hypophosphataemia [7] although albumin is not a nutritional marker. The biochemical abnormalities and associated symptoms seen in RFS are summarized (Table 1).

## 3. Management

The principles of management are to correct biochemical abnormalities and fluid imbalances returning levels to normal where possible. The optimum timing for correcting abnormalities in established RFS has been the source of controversy. The view that correction of electrolyte abnormalities must occur before commencement of feeding [1] has been revised and recent National Institute of Health and Clinical Excellence in the United Kingdom guidelines [29] indicate that feeding and correction of biochemical abnormalities can occur in tandem without deleterious effects to the patient [5, 8]. No published randomised trial data is available to support either view.

Prevention is the key to successful management [38]. Three factors appear fundamental: early identification of at risk individuals, monitoring during refeeding (Table 3), and an appropriate feeding regimen. Anticipating the risk of developing RFS prevents complications before they develop. This is aided by taking a detailed history, through clinical examination and by identifying high-risk patients with early involvement of the nutrition support team [39, 40]. Patients should be screened for risk of developing RFS on admission to hospital or when being assessed in the community. Those identified as being either malnourished or at high risk of not being able to meet their nutritional requirements should be appropriately referred for a formal nutritional assessment. Qualified dieticians or specialist nutrition nurses are required to perform nutritional assessments leading to the formulation of individualized strategies for the patient [16]. Effective communication within and between teams is a pre-requisite to achieve best care. The successful management of patients requires a multidisciplinary approach including nutritionists, nurses, and doctors meeting regularly to discuss changing nutritional needs of patients [39, 41–43].

## 4. Feeding Regimen in RFS

There are numerous published regimens for feeding patients at risk of RFS. None are evidence based. Irrespective of which particular feeding regimen is employed, the common denominator must be to follow the principles of permissive underfeeding. We recommend a regime (Table 4) based on current guidelines, published literature, and expert opinion [5, 29, 44, 45].

## 5. Summary

All clinicians caring for vulnerable groups who might require nutritional support should recognize the risk of RFS. The lack of randomized controlled trials in this area of medicine means that management is based on anecdotal data rather than evidence. This emphasizes the importance of minimizing risks of RFS by cautious reintroduction of feeding. We recommend that all patients receiving artificial nutritional support are entered into a database that can be regularly audited and evaluated to ensure best practice and adherence to current guidelines.

It is important to emphasize that RFS does not represent a singular condition or syndrome rather it describes an illness spectrum that occurs under particular circumstances within high-risk populations. Improved understanding of energetic requirements in healthy and sick patients will help improve understanding and allow for developing novel strategies to minimize risk of RFS to patients.

## Figures and Tables

**Table 1:** Clinical manifestations of electrolyte abnormalities associated with refeeding syndrome [1, 5–9].

	Clinical Manifestation
Phosphate (PO_4_^2−^)	Hypophosphataemia (normal range 0.8–1.45 mmol/l) presents as
	Cardiovascular: heart failure, arrhythmia, hypotension, cardiomyopathy shock, death
	Renal: acute tubular necrosis, metabolic acidosis
	Skeleton: rhabdomyolysis, weakness, myalgia, diaphragm weakness
	Neurology: delirium, coma, seizures, tetany
	Endocrine: hyperglycemia, insulin resistance, osteomalacia
	Haematology: haemolysis, thrombocytopenia, leukocyte dysfunction

Potassium (K^+^)	Hypokalemia (normal range 3.5–5.1 mmol/l) presents as
	Cardiovascular: hypotension, ventricular arrhythmias, cardiac arrest, bradycardia or tachycardia
	Respiratory: hypoventilation, respiratory distress, respiratory failure
	Skeleton: weakness, fatigue, muscle twitching
	Gastrointestinal: diarrhoea, nausea, vomiting, anorexia, paralytic ileus, constipation
	Metabolic: metabolic alkalosis

Magnesium (Mg^2+^)	Hypomagnesaemia (normal range 0.77–1.33 mmol/l) presents as
	Cardiovascular: paroxysmal atrial or ventricular arrhythmias, repolarisation alternans
	Respiratory: hypoventilation, respiratory distress, respiratory failure
	Neuromuscular: weakness, fatigue, muscle cramps (Trousseau and Chvostek) weakness, ataxia, vertigo, paresthesia, hallucinations, depression, convulsions
	Gastrointestinal: abdominal pain, diarrhoea, vomiting, loss of appetite, and constipation
	Other: anaemia, hypocalcemia
	NB: many cases of hypomagnesaemia do not manifest clinically till very late

Sodium (Na^+^)	Hyponatremia (normal range 136–145 mmol/l) ensues during RFS due to hyperglycaemia and presents as:
	Cardiovascular: heart failure and arrhythmia
	Respiratory: respiratory failure, pulmonary oedema.
	Renal: renal failure
	Skeleton: muscle cramps, fatigue, fluid retention and swelling (oedema)

Vitamins	Deficiency of thiamine (especially in alcoholism) presents as
	Neurology: Wernicke-Korsakoff syndrome, Karsakoff's psychosis,
	Cardiovascular: congestive heart failure and lactic acidosis, beriberi, disease
	Skeleton: muscle weakness

**Table 2:** Malnourished patients at risk of RFS [5, 6, 8, 29].

Anorexia nervosa	Chronic alcoholism
Radiation therapy	Major stressors without food for >7 days
Oncology patients	Postoperative patients
Severe malnutrition (Marasmus/Kwashiorkor)	Institutionalized patients
Pathological weight loss	Hunger strikes
Stroke (Neurological problems)	Malabsorption diseases
Inflammatory bowel diseases	Post bariatric surgery
Chronic pancreatitis	Elderly, poor social circumstance
Acquired Immunodeficiency Syndrome	Diabetes Mellitus

**Table 3:** Monitoring patients at risk of developing RFS [5, 6, 8, 30].

Clinical monitoring	Biochemical monitoring
Early identification of high risk patients	Monitor biochemistry and electrolyte levels
Monitor blood pressure and pulse rate	Monitor blood glucose levels
Monitor feeding rate	ECG monitoring in severe cases
Meticulously document fluid intake and output	Account other sources of energy
Monitor change in body weight	(dextrose, propofol, medications)
Monitor for neurologic signs and symptoms	
Patient education	

**Table 4:** Refeeding regime for patients at risk of RFS [5, 29].

Day	Calorie intake (All feeding routes)	Supplements
Day 1	10 kcal/kg/day For extreme cases (BMI < 14 kg/m^2^ or no food >15 days) 5 kcal/kg/day Carbohydrate: 50–60% Fat: 30–40% Protein: 15–20%	Prophylactic supplement PO_4_^2−^: 0.5–0.8 mmol/kg/day K^+^: 1–3 mmol/kg/day Mg^2+^: 0.3-0.4 mmmol/kg/day Na^+^: <1 mmol/kg/day (restricted) IV fluids-Restricted, maintain “zero” balance IV Thiamine + vitamin B complex 30 minutes prior to feeding

Day 2–4	Increase by 5 kcal/kg/day If low or no tolerance stop or keep minimal feeding regime	Check all biochemistry and correct any abnormality Thiamine + vitamin B complex orally or IV till day 3 Monitoring as required (Table 3)

Day 5–7	20–30 kcal/kg/day	Check electrolytes, renal and liver functions and minerals Fluid: maintain zero balance Consider iron supplement from day 7

Day 8–10	30 kcal/kg/day or increase to full requirement	Monitor as required (Table 3)

If RFS is suspected based on clinical and biochemical assessment or the patient develops intolerance to artificial nutritional support, the energetic intake should be reduced or stopped.

Feeding rate should be increased to meet full requirements for fluid, electrolytes, vitamins, and minerals if the patient is clinically and biochemically stable.
